# Association between smartphone addiction, suicidal ideation and suicide attempt: a systematic review

**DOI:** 10.7717/peerj.21386

**Published:** 2026-06-08

**Authors:** Yufeng Li, Jiang Li, Yang Song, Liping Cui, Jinxia Zhan, Xiaohong Sun, Wei Wang, Ping Pan, Nannan Li, Lin Song, Xiaoyu Sui, Fangyuan Wang, Yongling Chen

**Affiliations:** 1 Department of Psychological Clinic, Qingdao Hospital,University of Health and Rehabilitation Sciences (Qingdao Municipal Hospital), Qingdao, Shandong, China; 2 Department of Neurology, Qingdao Hospital, University of Health and Rehabilitation Sciences (Qingdao Municipal Hospital), Qingdao, Shandong, China; 3 Yantai Nurses School of Shandong, Yantai, Shandong, China; 4 Yantai Health Vocational College, Yantai, Shandong, China

**Keywords:** Suicidal ideation, Smartphone addiction, Systematic review, Suicide attempt

## Abstract

**Background:**

Smartphone addiction has been linked to adverse mental health outcomes, but its association with suicidal ideation (SI) and suicide attempt (SA) remains inconclusive. This systematic review synthesizes evidence on the relationship between smartphone addiction and SI/SA.

**Methods:**

We systematically searched PubMed, Embase, Web of Science, and the Cochrane Library from 1971 to August 2025 for peer-reviewed English-language studies examining smartphone addiction and SI or SA. Study quality was assessed using the Newcastle-Ottawa Scale.

**Results:**

Eleven cross-sectional studies met inclusion criteria. Nine studies were conducted in South Korea, one in China, and one in the United States. Most studies (7/11) were high quality. Significant positive associations between smartphone addiction and SI were consistently reported across adolescents, college students, and young adults. A dose-response relationship was observed, with high-risk users showing greater SI odds (OR range: 1.14–4.57). For SA, significant associations were also evident in high-risk groups (OR range: 1.15–1.87). The relationship was moderated by synergistic factors such as stress and usage duration.

**Conclusion:**

Smartphone addiction is significantly associated with increased odds of SI and SA across different populations. The relationship mostly exhibits a dose-response pattern, with higher addiction severity correlating with increased odds. These findings support considering problematic smartphone use in suicide prevention strategies across age groups.

## Introduction

In the era of information technology, smartphones have become an integral part of daily life worldwide, particularly among teenagers. Global smartphone users reached 2.1 billion in 2017 and this number exceeded 2.8 billion by 2020 (Meng et al., 2022). Smartphones hold particular appeal for individuals across nearly all age groups, especially adolescents and college students (Hartwell et al., 2024; Twenge, Martin & Campbell, 2018), who utilize these devices for academic tasks, internet browsing, social media engagement, and personal communication (Al-Mohaimeed, Alharbi & Mahmood, 2022; Oh & Park, 2022). While smartphones offer considerable convenience, their persistent and maladaptive use poses risks of addiction (Elhai et al., 2017; Yu & Sussman, 2020). This behavioral compulsion—also referred to as smartphone addiction, dependence, overuse, or problematic smartphone use—is characterized by significant impairments in physiological, psychosocial, or occupational functioning resulting from excessive engagement with the device (Choliz, 2010; Zhong et al., 2022). This phenomenon is considered an extension of internet overuse or internet addiction (Montag et al., 2021). Although a universally accepted definition remains debated, smartphone addiction is generally conceptualized as a behavioral addiction. It is defined by core dimensions such as salience, tolerance, a perceived lack of self-control, and functional impairment (Lee et al., 2014; Wu, Lin & Lin, 2021).

Extensive research has linked smartphone addiction to various adverse outcomes, including psychological distress, physiological complications, and impairments in academic and interpersonal functioning (Cheng et al., 2024; Geng & Liu, 2025; Guclu, Guclu & Demirci, 2024; Hong, Yeom & Lim, 2021). Studies have consistently associated smartphone overuse with increased risks of anxiety and depression (El-Sayed Desouky & Abu-Zaid, 2020; Elhai et al., 2020; Kim et al., 2018; Lee et al., 2016), particularly among university students (Demirci, Akgonul & Akpinar, 2015), young adults and individuals with lower educational attainment (Alhassan et al., 2018). Furthermore, smartphone addiction has been found to significantly impair sleep quality (Dibben et al., 2023) and reduce sleep duration (Kim et al., 2020b), thus increasing headache duration (Demir & Sumer, 2019). Additional physiological issues include neck and upper back pain (Ladeira et al., 2023), as well as wrist and hand pain (Al-Dhafer et al., 2023). It has also been linked to an unhealthy lifestyle characterized by overweight, decreased physical activity, and higher consumption of fast food (Alosaimi et al., 2016).

Suicidal behavior constitutes a major global public health burden, contributing to significant mortality and disability worldwide (Klonsky, May & Saffer, 2016). Suicide is the fifteenth leading cause of death globally, responsible for 1.4% of all deaths (WHO, 2014). Beyond fatal outcomes, 9.2% and 2.7% of the general population have experienced suicidal ideation (SI) and non-fatal suicide attempt (SA), respectively, at some point in their lives (Nock et al., 2008a). These behaviors are strong predictors of death by suicide and are associated with serious consequences such as physical injury, hospitalization, loss of personal freedom, and substantial economic costs (Nock et al., 2008b). There is an undeniable need to enhance the understanding of risk factors and prevention strategies related to suicidal thoughts and behaviors. Previous research has examined the relationship between smartphone addiction and SI across various age groups. Smartphone addiction is associated with an increased risk of SI among adolescents (Cha et al., 2023; Kim, 2022; Lee & Lee, 2023; Shinetsetseg et al., 2022) as well as college students and adults (Huang et al., 2021; Jeong et al., 2020; Kim et al., 2019; Noel et al., 2023). However, other studies have found no significant association, either in the general population (Sohn et al., 2018) or in specific subgroup analyses, such as those focused on gaming-related smartphone use (Lee et al., 2020), moderate addiction levels (Kim et al., 2020a), and usage duration of 4–8 h per day (Kim & Han, 2020). Similar inconsistencies have been observed regarding the association between smartphone addiction and SA.

In light of these conflicting findings, we conducted a systematic review to evaluate the association of smartphone addiction with both SI and SA. This study could help psychiatrists and educators to manage smartphone and related psychological issues for individuals across all age groups.

## Materials & Methods

### Study selection

The study was registered with PROSPERO (ID: CRD420251121276). A comprehensive literature search was performed in PubMed, Embase, Web of Science, and the Cochrane Library, covering publications from 1971 to August 2025. The aim was to identify peer-reviewed English-language studies that investigated the association between smartphone addiction and SI or SA. The detailed search strategies are provided in File S1. The full review protocol has not been publicly disseminated or posted online.

### Inclusion and exclusion criteria

We defined the study eligibility criteria according to the PECO framework: (a) Population: smartphone users; (b) Exposure: self-reported smartphone addiction; (c) Comparator: non-addicted smartphone users; and (d) Outcome: occurrence of SI or SA. Studies were excluded based on the following criteria: (a) non-primary literature (*e.g.*, case reports, reviews, guidelines, commentaries, or conference abstracts); (b) studies focusing on populations without smartphone addiction; (c) studies with no extractable quantitative data or insufficient outcome measures; or (d) publications not in English.

### Study selection, data collection, and data extraction

Data extraction was independently conducted by two investigators (Yufeng Li and Li Jiang) in August 2025, with both reviewers performing comprehensive screening of all eligible studies. Key study characteristics were synthesized into a summary table (Table 1) by Yang Song, which was then cross-checked and verified by Fangyuan Wang. Any discrepancies that arose during the review process were resolved by the senior author (Fangyuan Wang). Study quality was assessed using the Newcastle-Ottawa Scale (NOS) (Wells et al., 2000), with scores of 7–9 indicating high quality, 4–6 moderate quality, and 0–3 low quality.

**Table 1:** Main characteristics of the included studies examining the association among smartphone addiction, SI, and SA.

**Study**	**Study design**	**Origin**	**Measurement of exposure**	**Definition of exposure**	**Measurement of SI**	**Measurement of SA**	**Characteristics of participants**
Sohn et al. 2018	Cross-sectional study	Aisa	Smartphone addiction proneness scale	Potential risk group: scores of 41–43; high-risk group: scores of >44	Suicidal Ideation Questionnaire for High School Students (SIQ-HS)	–	416 students from 10th to 12th grade
Kim et al. 2019	Cross-sectional study	Aisa	Smartphone addiction proneness scale	High-risk group: scores of ≥44 or disturbance of adaptive functions domain score ≥15, withdrawal domain score ≥13 and a tolerance domain score ≥13; potential risk group: scores of 40–43, or disturbance of adaptive functions domain score ≥14	Single question (Have you ever thought about wanting to die in the last year?)	–	608 college students, predominantly 20 years old
Lee et al. 2020	Cross-sectional study	Aisa	Simple question	Smartphone overuse: use of a smartphone over 5h/day	Simple question (In the past year, did you ever seriously consider attempting suicide?)	–	62,276 Korean adolescents (aged 12–18 years)
Kim et al., 2020a	Cross-sectional study	Aisa	Self-administered, web-based survey	Low-use group: <16 h/week, moderate-use group: 16–29 h/week, high-use group: ≥30 h/week	Simple question (During the last 12 months, have you ever seriously thought about suicide?)	Simple question (During the last 12 months, have you ever attempted suicide?)	54,603 Korean middle and high school students
Kim & Han, 2020	Cross-sectional study	Asia	Simple question	“During the weekdays of the last 30 days, for how many hours on average did you use a smartphone in one day?” and “During the weekends of the last 30 days, for how many hours on average did you use a smartphone in one day?”	Simple question (During the past 12 months, have you ever seriously thought of committing suicide?)	Simple question (During the past 12 months, have you ever seriously thought of committing suicide?)	62,276 middle and high school students
Kim, 2022	Cross-sectional study	Aisa	Smartphone overdependence scale	Scores 23–30: potential risk, scores ≥31: high risk	Simple question (“In the past 12 months, have you seriously considered suicide?”)	Simple question (“In the past 12 months, have you attempted suicide?”)	25,987 high school students
Huang et al., 2021	Cross-sectional study	Asia	Mobile phone addiction index (MPAI) and self-reported daily use time	Daily use: hours of daily smartphone use dichotomized at >5 h *vs*≤5 h	Simple question (“Did you feel that life was not worth living in the past 1 year?”)	–	439 college students
Shinetsetseg et al., 2022	Cross-sectional study	Aisa	Smartphone overdependence scale	General user group/reference: <23 points, potential-risk user group: 23-30 points, high-risk user group: >31 points	Simple question (“At any time within the last 12 months, did you think about trying to kill yourself?”)	Simple question (“At any time within the last 12 months, did you ever attempt suicide?”)	54,948 Korean adolescents
Lee & Lee, 2023	Cross-sectional study	Aisa	Smartphone overdependence scale	General-use group: ≤22 points, potential-risk group: 23-30 points, high-risk group: ≥31 points	Simple question (“In the past 12 months, have you ever seriously considered suicide?”)	–	54,948 Korean adolescents
Cha et al., 2023	Cross-sectional study	Aisa	Self-answered questionnaire developed by the National Information Society Agency in 2016 and simple question	Questionnaire: cut-off ≥31 for high-risk group/smartphone overdependence Question: “Have you used your own or someone else’s smartphone in the last 7 days?” and “In the last 7 days, how many hours per day did you use the smartphone in average?” Answer: Yes	Suicidality assessed by asking whether participants had suicidal ideas, plans, or attempts in the past 12 months.	Suicidality assessed by asking whether participants had suicidal ideas, plans, or attempts in the past 12 months.	54,948 students from 793 middle and high schools
Noel et al., 2023	Cross-sectional study	America	Smartphone addiction scale-short version	Scores≥33 for women and ≥31 for men identified as smartphone addiction	Simple question (“During the past 12 months, did you ever seriously consider attempting suicide?”)	–	1,021 young adults living in Rhode Island for at least part of the year

**Notes.**

Abbreviations SI suicidal ideation OR odds ratio SNS Social-Network Services aOR/AOR adjusted OR PSU problematic smartphone use

## Results

### Characteristics of included studies

**Figure 1:**
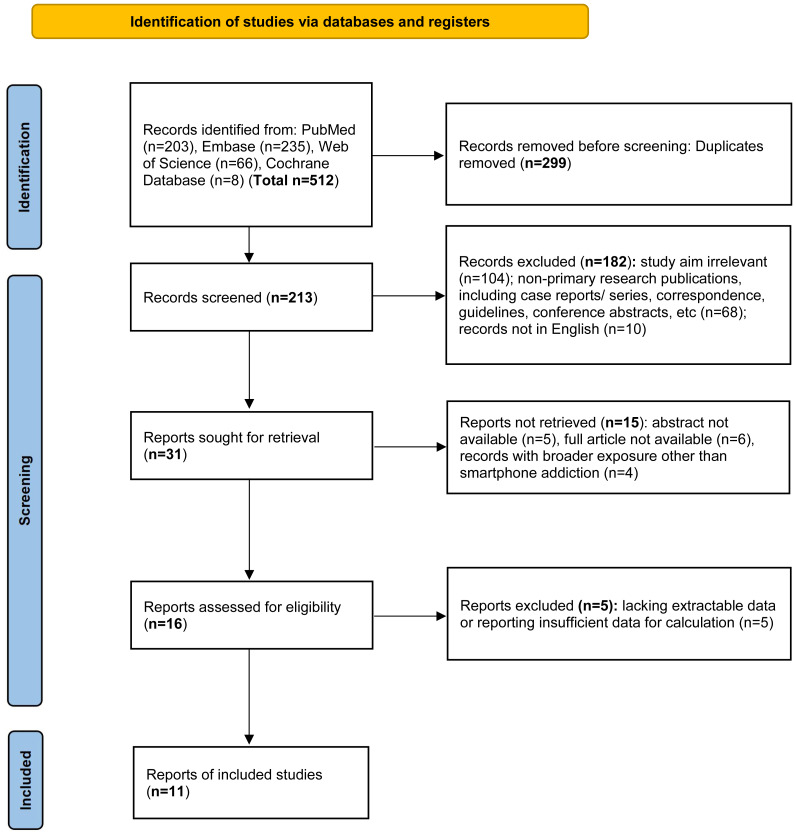
PRISMA flow diagram of the general selection process.

Figure 1 showed the flow diagram of the selection process. The literature search yielded 512 records, of which 299 duplicates were removed. The remaining 213 articles were assessed for relevance, with 182 being excluded due to study aim irrelevant, non-primary research publications, and records not in English. Subsequently, 16 articles were eligible for full-text screening, of which five articles were excluded due to lacking extractable data or reporting insufficient data for calculation. Ultimately, 11 cross-sectional studies were included to investigate the effect of smartphone addiction on SI (Cha et al., 2023; Huang et al., 2021; Kim, 2022; Kim et al., 2020a; Kim et al., 2019; Kim & Han, 2020; Lee et al., 2020; Lee & Lee, 2023; Noel et al., 2023; Shinetsetseg et al., 2022; Sohn et al., 2018) and five reported results of SA (Cha et al., 2023; Kim, 2022; Kim et al., 2020a; Kim & Han, 2020; Shinetsetseg et al., 2022). Table 1 outlines the key characteristics of the included studies. Nine studies were from South Korea, one from China, and one from the United States. The recruited population was mainly adolescents, middle and high school students, college students and adults. Identification of smartphone addiction included smartphone addiction proneness scale, self-answered simple question, smartphone overdependence scale, mobile phone addiction index (MPAI) and smartphone addiction scale. The measurement of SI and SA was mainly based on simple question with answer “yes” or “no”. The mean NOS score for the 11 studies was 6.3. Seven studies were high quality and four were moderate quality (Table 2).

**Table 2:** Quality assessment of the studies based on the Newcastle-Ottawa scale.

Study	Selection	Comparability	Outcome	Total
Sohn et al., 2018	★★★	–	★★★	5
Kim et al., 2019	★★★	★★	★★	7
Lee et al., 2020	★★★	★★	★★	7
Kim et al., 2020a	★★★	★★	★★	7
Kim & Han, 2020	★★★	★★	★★	7
Kim, 2022	★★★	★★	★★	7
Huang et al., 2021	★★★	–	★★	5
Shinetsetseg et al., 2022	★★★	–	★★	5
Lee & Lee, 2023	★★★	★★	★★	7
Cha et al., 2023	★★★	★★	★★	7
Noel et al., 2023	★★	–	★★★	5

### Association between smartphone addiction and SI

Across all included studies, most studies reported a consistent and significant positive association between smartphone addiction or overuse and SI among middle or high school students, adolescents and young adults, as shown in Table 3.

**Table 3:** Main findings of the included studies examining the association between smartphone addiction and SI.

**Study**	**Study design**	**Main findings**	**Adjusted confounding** **factors**
Sohn et al., 2018	Cross-sectional study	Smartphone addiction was significantly associated with SI (OR: 2.4, 95% CI [1.1–5.4]) in Model 2 (≥30 SIQ-HS), but not in Model 1 (≥3 SIQ-HS) and Model 3 (≥40 SIQ-HS).	None
Kim et al., 2019	Cross-sectional study	The ORs (95% CIs) of smartphone overuse in SI was estimated as 2.24 (1.52–3.31).	Age, sex, major, income, residence, smoking and alcohol drinking
Lee et al. 2020	Cross-sectional study	Compared to the “Study” group, the “SNS” group (AOR 4.57; 95% CI [4.20–4.98]), “Game” group (AOR 2.24; 95% CI [2.05–2.45]) and “Entertainment” group (AOR 2.60; 95% CI [2.40–2.81]) were significantly to report SI.	Age, sex, region of residence, family economic status, sleep hours, and physical activity
Kim et al., 2020a	Cross-sectional study	Compared with low-use group: Moderate-use: OR = 1.00 (95% CI [0.92–1.09]), high-use: OR = 1.14 (95% CI [1.05–1.24])	Age, sex, economic status, living apart from family, behavioral characteristics, conflict with family, conflict with peers, and disturbance in school work
Kim & Han, 2020	Cross-sectional study	1. Compared with smartphone use for education: Use for communication: aOR = 1.05 (95% CI [0.93–1.19]), use for enjoyment: aOR = 1.11 (95% CI [0.98–1.25]). 2. Compared with number of hours spent using a smartphone on weekdays (<2 h/day): >2, <4: aOR = 0.94 (95% CI [0.87–1.02]), ≥4: aOR = 1.06 (95% CI [0.98–1.15]). 3. Compared with number of hours spent using a smartphone on weekends (<4 h/day): >4, <8 aOR=1.00 (95% CI [0.93–1.07]), ≥8: aOR = 1.22 (95% CI [1.12–1.32])	Gender, school year, perceived academic record, family structure, parental educational level, perceived economic status, place of residence, stress, and depressive symptoms
Kim, 2022	Cross-sectional study	Smartphone overdependence and SI: 1. Individual effect: AOR = 1.44 (95% CI [1.32–1.57]) 2. Combined effects (reference: no overdependence+no stress): No overdependence+some stress: AOR = 2.10 (95% CI [1.64–2.68]) Overdependence+some stress: AOR = 3.33 (95% CI [2.60–4.25]) No overdependence+high stress: AOR = 8.85 (95% CI [7.03–11.14]) Overdependence+high stress: AOR = 12.58 (95% CI [9.97–15.88])	Grade and gender, residential area, socioeconomic status, academic achievement, perceived health status, smoking, drinking, substance abuse, sexual intercourse, physical activity, and sleep quality
Huang et al., 2021	Cross-sectional study	SI was significantly correlated with more than 5 h of daily smartphone use. Daily smartphone use > 5 h *vs*≤5 h: OR = 2.60 (95% CI [1.13–6.01])	None
Shinetsetseg et al., 2022	Cross-sectional study	Compared with general user group: Potential-risk group: OR = 1.50 (95% CI [1.41–1.60]), high-risk group: OR = 2.49 (95% CI [2.21–2.81])	None
Lee & Lee, 2023	Cross-sectional study	PSU and suicidal ideation: Girls: aOR = 1.77 (95% CI [1.64–1.91]) Boys: aOR = 1.82 (95% CI [1.66–1.99]) PSU prevalence: 22.1% potential-risk, 3.0% high-risk, SI prevalence: 16.8% in PSU group, 7.9% in non-PSU group	Sex, age, area of residence, school grade, household income level, academic achievement, BMI, and cigarette and alcohol use
Cha et al., 2023	Cross-sectional study	Usage >4 h/day *vs* <4 h/day: SI: OR = 1.22 (95% CI [1.13–1.31]) Suicidal plan: OR = 1.17 (95% CI [1.04–1.31]) Smartphone usage >4 h/day was significantly associated with SI.	Age, sex, residence, socioeconomic status, and academic achievement
Noel et al., 2023	Cross-sectional study	In the unadjusted analysis, smartphone addiction was significantly associated with the odds of suicide ideation (OR [95% CI] 1.55 [1.08–2.20]).	None

**Notes.**

Abbreviations SI suicidal ideation OR odds ratio SNS Social-Network Services aOR/AOR adjusted OR PSU problematic smartphone use

**Middle or high school students:** Sohn et al. (2018), in a study of 416 Korean high school students, reported that smartphone addiction was significantly associated with SI (OR = 2.4, 95% CI [1.1–5.4]) in Model 2 (≥30 Suicidal Ideation Questionnaire for High School Students (SIQ-HS)), but not in Model 1 (≥3 SIQ-HS) and Model 3 (≥40 SIQ-HS). Using data from the 2017 Korea Youth Risk Behavior Web-based Survey (KYRBWS), Kim et al. (2020a) found that compared with the low smartphone-use group, the high smartphone-use groups (OR = 1.14, 95% CI [1.05–1.24]) had a higher odds of suicidal thoughts after adjusting for covariates, while the odds for moderate group only showed trends of higher risk (OR = 1.00, 95% CI [0.92–1.09]). Another study conducted by Kim & Han (2020) also used data from the 2017 KYRBWS and found that smartphone use for communication purposes showed positive tendency of SI (OR = 1.05, 95% CI [0.93–1.19]). Similar trends were observed among groups with smartphone use of >2 to <4 h/day on weekdays and >4 to <8 h/day on weekends. The association was significant for smartphone using time ≥8 h/day for weekends (OR = 1.22, 95% CI [1.12–1.32]). Kim (2022) further elucidated this relationship by demonstrating a combined effect. While smartphone overdependence alone showed a powerful relationship with SI (OR = 1.44, 95% CI [1.32–1.57]), the combination of high stress and smartphone overdependence yielded the more significant association (OR = 12.58, 95% CI [9.97–15.88]). Another study with KYRBWS by Cha et al. (2023) demonstrated that smartphone usage >4 h/day was significantly associated with all suicidal outcomes, including SI (OR = 1.22, 95% CI [1.13–1.31]) and suicidal plan (OR = 1.17, 95% CI [1.04–1.31]).

**Adolescents:** Lee et al. (2020) found that, compared to the “Study” group, the “Social-Network Services (SNS)” group (OR 4.57, 95% CI [4.20–4.98]), “Game” group (OR 2.24, 95% CI [2.05–2.45]) and “Entertainment” group (OR 2.60, 95% CI [2.40–2.81]) were significantly to report SI outcome in Korean adolescents. Similarly, Shinetsetseg et al. (2022) also demonstrated a dose–response relationship, where the risk of SI increased with the severity of smartphone addiction. Compared to general users, the OR for SI was 1.50 (95% CI [1.41–1.60]) for the potential-risk group and 2.49 (95% CI [2.21–2.81]) for the high-risk group.

**College students:** In a study of 608 Korean college students, Kim et al. (2019) reported that individuals with smartphone overuse had a significantly higher likelihood of experiencing SI. The OR for SI in the smartphone overuse group was 2.24 (95% CI [1.52–3.31]). Huang et al. (2021), in a study of 439 Chinese college students, found that excessive daily smartphone use (more than 5 h) was significantly associated with SI (OR = 2.60, 95% CI [1.13–6.01]). However, only the relationship between SI and excessive smartphone use, but not the MPAI score itself, was significant, suggesting that usage time may be a more critical factor in this population. Lee & Lee (2023) reported this association stratified by sex. Both girls (OR = 1.77, 95% CI [1.64–1.91]) and boys (OR = 1.82 95% CI [1.66–1.99]) with problematic smartphone use (PSU) had higher odds of SI than non-PSU groups.

**Young adults:** Noel et al. (2023), in a sample of 1,021 U.S. young adults, reported that those with smartphone addiction had 1.55 times higher odds (95% CI [1.08–2.20]) of experiencing SI in the past year, even after adjusting for sociodemographic factors.

### Association between smartphone addiction and SA

The main findings regarding SA were listed in Table 4.

**Table 4:** Main findings of the included studies examining the association between smartphone addiction and SA.

**Study**	**Study design**	**Main findings**	**Adjusted confounding** **factors**
Kim et al., 2020a	Cross-sectional study	Compared with low-use group: Moderate-use: OR = 0.96 (95% CI [0.79–1.17]), high-use: OR = 1.32 (95% CI [1.10–1.59])	Age, sex, economic status, living apart from family, behavioral characteristics, conflict with family, conflict with peers, and disturbance in school work
Kim & Han, 2020	Cross-sectional study	1. Compared with smartphone use for education: Use for communication: aOR = 0.91 (95% CI [0.73–1.13]), use for enjoyment: aOR = 0.94 (95% CI [0.76–1.17]). 2. Compared with number of hours spent using a smartphone on weekdays (<2 h/day): >2, <4: aOR=0.85 (95% CI [0.72–0.98]), ≥4: aOR = 1.15 (95% CI [0.98–1.35]). 3. Compared with number of hours spent using a smartphone on weekends (<4 h/day): >4, <8 aOR = 0.83 (95% CI [0.72–0.96]), ≥8: aOR = 1.28 (95% CI [1.10–1.49])	Gender, school year, perceived academic record, family structure, parental educational level, perceived economic status, place of residence, stress, and depressive symptoms
Kim, 2022	Cross-sectional study	Combined effects: The combination of high stress and smartphone overdependence showed the highest odds for SA (AOR: 5.47, 95% CI [3.09–9.68]). In the absence of stress, smartphone overdependence was not associated with an increased risk of SA (AOR: 1.10, 95% CI [0.90–1.34]).	Grade and gender, residential area, socioeconomic status, academic achievement, perceived health status, smoking, drinking, substance abuse, sexual intercourse, physical activity, and sleep quality
Shinetsetseg et al., 2022	Cross-sectional study	Compared with general user group: Potential-risk group: OR = 1.10 (95% CI [0.96–1.27]), high-risk group: OR = 1.87 (95% CI [1.48–2.37])	None
Cha et al., 2023	Cross-sectional study	Usage >4 h/day *vs* <4 h/day: SA: OR = 1.20 (95% CI [1.03–1.40]) Smartphone usage >4 h/day was significantly associated with SA.	Age, sex, residence, socioeconomic status, and academic achievement

**Notes.**

Abbreviations SA suicide attempt OR odds ratio aOR/AOR adjusted OR

**Middle or high school students:** Results from Kim et al. (2020a) showed that the high smartphone-use group had higher odds of SA compared to the low smartphone-use group (OR = 1.32, 95% CI [1.10–1.59]). Another study by Kim & Han (2020) demonstrated that smartphone use of ≥8 h/day on weekends was significantly associated with SA (OR = 1.28, 95% CI [1.10–1.49]). In contrast, smartphone use of >2 to <4 h/day on weekdays and >4 to <8 h/day on weekends was associated with lower odds of SA relative to the respective control groups. Kim (2022) found that the combination of high stress and smartphone overdependence yielded the highest odds for SA (OR = 5.47, 95% CI [3.09–9.68]), whereas smartphone overdependence alone was not associated with SA in the absence of stress (OR = 1.10, 95% CI [0.90–1.34]). Additionally, a study by Cha et al. (2023) showed that smartphone use exceeding 4 h/day was significantly associated with suicidal outcomes, specifically SA (OR = 1.20, 95% CI [1.03–1.40]).

**Adolescents:** Adolescents exhibited heightened vulnerability, with excessive smartphone use compounding the impact of psychosocial stressors on SI and behavior; this association remained significant even after adjusting for depression, anxiety, and sleep disturbances. Shinetsetseg et al. (2022) demonstrated that, compared to general users, the OR for SA was 1.10 (95% CI [0.96–1.27]) for the potential-risk group and 1.87 (95% CI [1.48–2.37]) for the high-risk group, indicating that adolescents were also vulnerable when facing smartphone overuse.

### Role of content type and duration of smartphone use

**Content type:** Two studies provided insights into how the purpose of smartphone use influenced its association with SI and SA. Lee et al. (2020) explored the association between different types of smartphone use and SI, concluding that using smartphones for SNS, gaming, and entertainment was associated with higher odds of SI. In a study by Kim & Han (2020), the odds associated with smartphone use for education, communication, and enjoyment were comparable, suggesting that the higher odds of SI may not be related to the content type of smartphone use. Similar results were reported for SA in the same study.

**Duration:** Five studies reported a time- or degree-dependent effect of smartphone addiction or overuse. Although the cutoffs varied across studies, groups with longer use time or classified as high-use or high-risk consistently showed greater odds of SA compared to those with shorter use time or classified as low-use or low-risk.

## Discussion

Our study provides evidence consistently supporting a positive association between smartphone addiction and SI across different populations. The relationship often follows a dose–response pattern, with higher addiction severity correlating with greater risk. The association with SA is also significant, though it may be more evident in high-risk groups. These findings highlight the importance of addressing problematic smartphone use as a potential risk factor or component of mental health prevention for different populations.

While the underlying mechanisms linking smartphone overuse to an increased risk of SI and SA remain not fully understood, several potential explanations have been proposed. First, excessive smartphone use is associated with sleep disturbances, largely due to delayed bedtimes, which may in turn contribute to increased stress and depression (Chen et al., 2017). Sleep disturbance itself is associated with heightened SI (Cohen et al., 2024), as evidenced in general adolescent populations (Gowin et al., 2024), Chinese undergraduate students (Wu et al., 2022), depressed youth (Fan et al., 2024). Second, individuals with prolonged smartphone use tend to engage in fewer face-to-face interactions and report lower levels of social support compared to those without smartphone overuse (Chen et al., 2017). Inadequate or diminishing social support has been linked to an increased risk of subsequent SI (Richie et al., 2021; Wang et al., 2022). Third, sedentary behaviors associated with smartphone use may serve as a significant predictor of adverse mental health outcomes among adolescents (Schuch et al., 2018). Fourth, gamma-aminobutyric acid (GABA) levels have been found to be significantly higher in the smartphone addiction group compared to the control group (Seo et al., 2020). Disruptions in neural signal regulation resulting from elevated GABA concentrations are associated with deficits in cognitive and emotional processing, which may trigger a cascade of effects ranging from drowsiness to more severe clinical manifestations such as anxiety and depressive disorders (Lener et al., 2017). In our systematic review, smartphone addiction was closely associated with an increased likelihood of SI and SA across all populations. Only one study examined the combination of high stress and smartphone overdependence, which was associated with a notably stronger effect. However, further studies employing mechanistic designs are warranted to validate these associations and elucidate the underlying pathways.

A concern raised in this review is that smartphone addiction, SI, and SA may all be associated with depression and psychological distress. We acknowledge that these factors represent important confounding variables that require careful consideration. Accumulating evidence consistently demonstrates that depression and stress serve as critical mediators in the relationship between smartphone addiction (Lei et al., 2020; Li et al., 2020; Nikolic et al., 2023) and suicide-related outcomes (Conejero et al., 2018; Patalay & Fitzsimons, 2021; Ribeiro et al., 2018). Furthermore, alternative pathways may explain this relationship. Lifestyle risk behaviors (*e.g.*, sleep deprivation, reduced physical activity) have been shown to mediate the association between smartphone overdependence and suicidal behaviors in adolescent populations (Hu et al., 2022). These findings suggest that smartphone addiction may be associated with suicide outcomes through distinct pathological mechanisms—such as impulse control deficits, maladaptive reality adaptation, and social isolation—that operate independently of depression. In our systematic review, only one study controlled for depressive symptoms, while two studies adjusted for sleep duration or sleep quality. Additionally, one study accounted for other psychological factors, including family and peer conflict, as well as academic impairment.

In our review, the significant association among smartphone addiction, SI and SA was consistently observed across middle and high school students, adolescents, college students, and young adults. The overall median prevalence of problematic smartphone use among children and young people is 23.3% (range: 14.0–31.2%) (Sohn et al., 2019). In a study of Swiss students, the prevalence was 16.9%, with smartphone addiction being more common among younger adolescents (aged 15–16 years), students with both parents born abroad, and those reporting lower levels of physical activity or higher levels of stress, compared to their older counterparts (aged 19 years and older) (Haug et al., 2015). Regarding SI, SI and death-related ideation were reported in 18.05% and 16.90% of children aged 6–12, respectively (Li et al., 2023), which is not uncommon. Even in a meta-analysis, the pooled prevalence estimates for SI and suicide planning were 7.5% and 2.2%, respectively, among children aged 12 years and younger (Geoffroy et al., 2022). Similar findings have been reported in other studies across various populations, including children and adolescents (Lim et al., 2019), preadolescent children (Lawrence et al., 2021), school learners aged 12–17 years (Quarshie, Dey & Oppong Asante, 2023), medical students (Uteh et al., 2022), college students (Li et al., 2014), adults (Liu et al., 2022; Omary, 2021). Notably, in our review, both adolescents and college students or adults were identified as high-risk populations for smartphone addiction, regardless of age range.

The limitations of this research encompass several key aspects. The non-randomized nature of the available evidence prevents causal inference regarding smartphone addiction, SI, and SA. Furthermore, the findings may be influenced by unmeasured confounders, including genetic variations, lifestyle-related factors, and the exclusion of non-English publications. Moreover, included studies of our review could not adjust for several covariates that may affect adolescents’ screen time, such as periods of unsupervised time at home, parental occupational status, and the quality of familial relationships. Additionally, the high heterogeneity observed during the meta-analysis further highlights variations across aspects of the topic, including the identification of smartphone addiction and suicidal outcomes, as well as factors such as age, presence of depressive symptoms, and stress.

## Conclusions

The current study revealed that excessive smartphone use is positively associated with the occurence of SI and SA within the general population, encompassing adolescents as well as college students and adults. The association might be dose-dependent, with longer using time displaying more increased odds. These findings underscore the importance of incorporating problematic smartphone use into mental health prevention and intervention strategies for diverse populations.

## Supplemental Information

10.7717/peerj.21386/supp-1 Supplemental Information 1 PRISMA checklist

10.7717/peerj.21386/supp-2 Supplemental Information 2 The search strategies, including key words

10.7717/peerj.21386/supp-3 Supplemental Information 3 Raw data

## Additional Information and Declarations

### Data Availability

The following information was supplied regarding data availability: This is a Systematic Review/Meta-analysis.
