# Supplementary material for: Association between smartphone addiction, suicidal ideation and suicide attempt: a systematic review
Source: PeerJ. 2026 Jun 8;14:e21386. doi: 10.7717/peerj.21386 (PMC13256117; doi:10.7717/peerj.21386)
Supplement: Supplemental Information 2 [file peerj-14-21386-s002.docx]

**Supplementary File 1 Search strategies**

1

("smartphone"[All Fields] AND "addiction"[All Fields]) OR "smartphone addiction"[All Fields]) AND ("suicidal ideation"[MeSH Terms] OR ("suicidal"[All Fields] AND "ideation"[All Fields]) OR "suicidal ideation"[All Fields])

2

("smartphone"[MeSH Terms] OR "smartphone"[All Fields] OR "smartphones"[All Fields] OR "smartphones"[All Fields]) AND ("overuse"[All Fields] OR "overused"[All Fields] OR "overuser"[All Fields] OR "overusers"[All Fields] OR "overuses"[All Fields] OR "overusing"[All Fields]) AND ("suicidal ideation"[MeSH Terms] OR ("suicidal"[All Fields] AND "ideation"[All Fields]) OR "suicidal ideation"[All Fields])

3

("smartphone"[All Fields] AND "addiction"[All Fields]) OR "smartphone addiction"[All Fields]) AND (("suicid"[All Fields] OR "suicidal ideation"[MeSH Terms] OR ("suicidal"[All Fields] AND "ideation"[All Fields]) OR "suicidal ideation"[All Fields] OR "suicidality"[All Fields] OR "suicidal"[All Fields] OR "suicidally"[All Fields] OR "suicidals"[All Fields] OR "suicide"[MeSH Terms] OR "suicide"[All Fields] OR "suicides"[All Fields] OR "suicides"[All Fields] OR "suicided"[All Fields] OR "suiciders"[All Fields]) AND ("attempt"[All Fields] OR "attempted"[All Fields] OR "attempter"[All Fields] OR "attempters"[All Fields] OR "attempting"[All Fields] OR "attempts"[All Fields]))

4

("smartphone"[MeSH Terms] OR "smartphone"[All Fields] OR "smartphones"[All Fields] OR "smartphone s"[All Fields]) AND ("overuse"[All Fields] OR "overused"[All Fields] OR "overuser"[All Fields] OR "overusers"[All Fields] OR "overuses"[All Fields] OR "overusing"[All Fields]) AND (("suicid"[All Fields] OR "suicidal ideation"[MeSH Terms] OR ("suicidal"[All Fields] AND "ideation"[All Fields]) OR "suicidal ideation"[All Fields] OR "suicidality"[All Fields] OR "suicidal"[All Fields] OR "suicidally"[All Fields] OR "suicidals"[All Fields] OR "suicide"[MeSH Terms] OR "suicide"[All Fields] OR "suicides"[All Fields] OR "suicide s"[All Fields] OR "suicided"[All Fields] OR "suiciders"[All Fields]) AND ("attempt"[All Fields] OR "attempted"[All Fields] OR "attempter"[All Fields] OR "attempters"[All Fields] OR "attempting"[All Fields] OR "attempts"[All Fields]))

5

("smartphone"[All Fields] AND "addiction"[All Fields]) OR "smartphone addiction"[All Fields]) AND ("suicidal ideation"[MeSH Terms] OR ("suicidal"[All Fields] AND "ideation"[All Fields]) OR "suicidal ideation"[All Fields] OR ("suicidal"[All Fields] AND "thoughts"[All Fields]) OR "suicidal thoughts"[All Fields])

6

("smartphone"[MeSH Terms] OR "smartphone"[All Fields] OR "smartphones"[All Fields] OR "smartphones"[All Fields]) AND ("overuse"[All Fields] OR "overused"[All Fields] OR "overuser"[All Fields] OR "overusers"[All Fields] OR "overuses"[All Fields] OR "overusing"[All Fields]) AND ("suicidal ideation"[MeSH Terms] OR ("suicidal"[All Fields] AND "ideation"[All Fields]) OR "suicidal ideation"[All Fields] OR ("suicidal"[All Fields] AND "thoughts"[All Fields]) OR "suicidal thoughts"[All Fields])
